# Transfection of unmodified oligodeoxynucleotide with polyethylenimine reduces the level of hepatitis B surface antigen

**DOI:** 10.3389/fmicb.2025.1600679

**Published:** 2025-05-01

**Authors:** Junyu Lin, Jing Li

**Affiliations:** ^1^Research Center for Basic Medical Science, Qilu Hospital of Shandong University, Jinan, Shandong, China; ^2^Department of Gastroenterology, Qilu Hospital of Shandong University, Jinan, Shandong, China

**Keywords:** hepatitis B virus infection, nonviral gene vector, unmodified oligonucleotide delivery, HBsAg secretion, anti-HBV treatment

## Abstract

**Introduction:**

The delivery of nucleic acid into cells using polyethylenimine (PEI) as non-viral carrier is a potential candidate technique for the treatment of hepatitis B virus (HBV) infection.

**Methods:**

In the present study, PEI was used as cationic polymers and transfected with unmodified oligodeoxynucleotides in cell cultures and the BALB/c mouse model to investigate its efficiency in blocking HBV surface antigen (HBsAg) secretion.

**Results and discussion:**

PEI/oligonucleotide complexes selectively inhibited HBsAg secretion in the culture supernatant, while there were no evident alterations in HBeAg and HBV DNA levels, thereby suggesting its potential inhibitory activity against the production of HBsAg. The complexes formed by PEI with double-stranded decoy oligonucleotides also suppressed HBsAg secretion but showed no expected interference with the intermediate levels of HBV transcription or replication. Furthermore, PEI/plasmid-DNA complexes demonstrated no influence on the expression levels of HBsAg, thus highlighting the specific effects of PEI/oligonucleotides exerted on HBsAg release. PEI-oligonucleotides transfection prior to the viral inoculation impaired HBV infection in HepG2-NCTP cells. Importantly, the PEI/oligonucleotide complex also induced the decline of HBsAg in hydrodynamically injected BALB/c mice. These findings demonstrate that transfection of PEI/oligonucleotide complexes can help effectively reduce HBsAg level and may offer a new potential avenue for the development of anti-HBV treatment.

## 1 Introduction

Approximately 290 million people worldwide present with chronic hepatitis B and are therefore at a risk of death owing to the complications of the disease, such as liver cirrhosis and hepatocellular carcinoma. Chronic hepatitis B is caused by the hepatitis B virus (HBV) (Yuen et al., 2018; Ganem and Prince, 2004). In HBV carriers, HBV-infected cells produce a substantial amount of non-infectious subviral particles (SVPs), which typically outnumber Dane particles by 1,000:1 to 10,000:1. SVPs contain only envelope glycoproteins such as HBV surface antigen (HBsAg), while Dane particles represent complete hepatitis B virions. The excessive production of SVPs and host-derived lipids can result in the development of immune tolerance and can contribute to the persistence of HBV infection (Kondo et al., 2013). However, the complete clearance of HBsAg from the bloodstream, which is called HBV functional cure, is rarely achieved with adoption of the current antiviral strategies such as nucleos (t) ides and interferon-based therapies (Cornberg et al., 2019; Tout et al., 2020). This highlights the need for developing more effective therapeutic agents to combat HBV infection.

Oligonucleotide-based therapy is emerging as a potential treatment modality for viral infections, tumors, and hereditary diseases. Different types of therapeutic oligonucleotides such as antisense oligonucleotide, small interference RNA (siRNA), anti-gene oligonucleotide, aptamer, decoy, and CpG oligonucleotides have been developed in the last few decades (Roberts et al., 2020; Hammond et al., 2021). Another type of therapeutic oligonucleotides is the nucleic acid polymer (NAP), a phosphorothioated single-stranded oligonucleotide, which demonstrates a broad-spectrum antiviral activity against several enveloped viruses (Vaillant, 2016). Its inhibition of secretion of HBsAg is dependent on the length and phosphorothioate moiety. However, it is independent of oligonucleotide sequences (Blanchet et al., 2019; Guillot et al., 2017).

Oligonucleotide-based drugs have promising applications in the clinical setting. An important requirement for their efficient use is their successful delivery into the cells. Several nonviral vectors for gene delivery using polycationic materials were reported because polycationic materials could be readily modified by adopting principles of rich polymer chemistry and synthetic methods (Yin et al., 2014). Polyethylenimine (PEI) is the gold standard for the cationic-polymeric delivery vector because it shows high transfection efficiency in various cell types (Pandey and Sawant, 2016). Additionally, PEI has been widely investigated in animal models and human subjects (Yan et al., 2014; Patnaik and Gupta, 2013). Owing to the cooperative electrostatic interaction between the ammonium groups of the PEI and the phosphate groups of the nucleic acid, PEI with high positive charge density spontaneously forms inter-polyelectrolyte complex (polyion complex) with nucleic acids. This results in the formation of PEI-oligonucleotide complexes that are similar to nanoparticles allowing efficient cellular uptake through endocytosis. PEI acts as a proton sponge that buffers low pH in the endolysosomal compartments, confers protection to the genes against degradation, and potentially induces endolysosomal membrane rupture, which releases the PEI/DNA complexes into the cytoplasm (Pandey and Sawant, 2016). Although several physicochemical properties of these complexes remain unknown, the PEI/plasmid-DNA complex shows higher stability than its PEI/ON counterpart because of the difference in the charges harbored by the pDNA and oligonucleotides (Ziebarth et al., 2017). Various studies suggested that although PEI served as an excellent non-viral gene transfection agent, it also presented with few toxicity issues. The toxicity issues related to PEI were correlated to its molecular weight and structure (branched PEI or liner PEI). The toxicity was more severe in the free PEI. However, its toxicity decreased after its association with oligonucleotides. Endocytic uptake of PEI caused endosomal swelling and rupture, which resulted in intracellular stress and mitochondrial alterations, thereby leading to apoptotic cell death, especially at higher doses of PEI (Pandey and Sawant, 2016).

In the current gene therapy strategies, the application of polyplexes consisting of PEI and DNA or siRNA has attracted attention. Therefore, in the present study, we aimed to investigate the antiviral activity of PEI/nucleotide complexes on HBsAg secretion, HBV gene expression and replication in HepAD38 cells and in a BALB/c mouse model and to determine the potential entry inhibition effect in HepG2-NTCP cells.

## 2 Materials and methods

### 2.1 Cell culture

HepAD38 cells were maintained in the Dulbecco’s modified Eagle’s medium F12 (DMEM/F12) complemented with 10% fetal bovine serum (FBS), 2 mM L-glutamine, 100 U/mL penicillin, 100 mg/mL streptomycin, and 100 μg/mL G418. HepG2-NTCP cells were maintained in DMEM with 10% FBS, 2 mM L-glutamine, 100 U/mL penicillin, 100 mg/mL streptomycin supplemented with 2 ug/mL puromycine (Gbico) and 2.5% DMSO. All cells were cultured in a humidified atmosphere with 5% CO_2_ at 37°C.

### 2.2 Commercial reagents

Branched PEI (MW 25 kDa, 1 ug/uL) and Dimethyl Sulfoxide (D2650) were purchased from the (Sigma Aldrich, USA). The X-tremeGENE 9 DNA Transfection Reagent (06365779001) was purchased from Roche (Mannheim, Germany). One-step DNA Transfecter (LD210-005) was used (Shanghai ENLIGHTEN Bioscience & Technology Co. Ltd., Shanghai, China). Oligodeoxynucleotides, including the fluorescein isothiocyanate (FITC)-labeled oligonucleotides (with different nt-length listed in Table 1), were synthesized (Shanghai Biosune Co. Ltd., Shanghai, China) and were uniformly resolved in nuclease-free water (Cell Signaling Technology, USA) at an initial concentration of 100 uM. The Opti-MEM™ I Reduced Serum Medium (#31985062, Gibco, USA) was a modification of the Eagle’s minimum essential medium and was used as an ideal medium for cationic polymer transfections. The plasmid pCMV-EGFP (pEGFP-C2, 4,735 bp) encoding enhanced green fluorescent protein (EGFP) under cytomegalovirus (CMV) promoter was previously obtained from the BD Biosciences Clontech (USA). Plasmid pHBV1.3 (#AF100309.1) was a generous gift from Dr. Zhongliang Shen (Fudan University, China). Polyclonal rabbit anti-Hepatitis B virus core antigen (HBc) antibody (B0586) was purchased from the Dako (Glostrup, Denmark), polyclonal rabbit anti-HBsAg antibody (NB100-62652) from Novus Biological (Littleton, CO, USA) and β-actin (AF0003) was purchased from the Beyotime Biotechnology (Shanghai, China). Anti-Rabbit IgG (H + L) cross-adsorbed secondary antibody (A-11010) was purchased from Invitrogen. The CCK-8 kit (#40203ES76), MTT Cell Proliferation assay Kit (#40206ES76) and Hoechst 33258 (#40730ES03) were purchased from Shanghai YiSheng biotech Co. Ltd. (Shanghai, China).

**Table 1:** Oligonucleotides used in the study.

Name	Sequence	Length (nt)	Molecular Weight (g/mole)
Unmodified single strand oligonucleotides			
10ntAC	5′- ACACACACAC -3’	10	2949.99
20ntAC	5′- ACACACACACACACACACAC -3’	20	5961.94
30ntAC	5′- ACACACACACACACACACACACACACACAC -3’	30	8973.89
40ntAC	5′- ACACACACACACACACACACACACACACACACACACACAC -3’	40	11985.84
Decoy oligonucleotides			
scramble-decoy-F	5′- GACTGACTGACTGACTGACTGACT −3’	24	7352.84
scramble-decoy-R	5′- AGTCAGTCAGTCAGTCAGTCAGTC -3’	24	7352.84
*Sp1*-decoy-1-F	5′- CCTTTTGGGGTGGAGCCCTCCCTTTTGGGGTGGAGCCCTC -3’	40	12293.96
*Sp1*-decoy-1-R	5′- GAGGGCTCCACCCCAAAAGGGAGGGCTCCACCCCAAAAGG −3’	40	12,304
*Sp1-*decoy-2-F	5′- TCCGCCTCCTGTCCGCCTCCTGTCCGCCTCCTG −3’	33	9856.34
*Sp1*-decoy-2-R	5′- CAGGAGGCGGACAGGAGGCGGACAGGAGGCGGA −3’	33	10417.79
*CREB*-decoy-F	5′- TGACGCAATGACGCAATGACGCAA −3’	24	7379.87
*CREB*-decoy-R	5′- TTGCGTCATTGCGTCATTGCGTCA −3’	24	7325.81
*HNF1α*-decoy-F	5′- AGTTAATCATTACTAGTTAATCATTACT −3’	28	8535.68
*HNF1α*-decoy-R	5′- AGTAATGATTAACTAGTAATGATTAACT −3’	28	8633.76

Sp1, Specificity protein 1; HNF1α, hepatocyte nuclear factor 1α; CREB, C-AMP-response element binding protein.

### 2.3 Preparation of PEI/ON complexes for transfection and HBV infection

The cells were evenly seeded in a 12-well plate at a density of 5 × 10^5^ cells per well and incubated for 24 h before transfection. Different mass concentrations of plasmid DNA or oligonucleotides (100 uM) were diluted in 120 uL of opti-MEM and then mixed with 2 uL of PEI (1 ug/uL) according to the different ratios for 15 min at room temperature. These transfection mixtures were added with 380 uL of serum-free DMEM in wells and incubated for duration of 5 h. After the performance of transfection, the transfection mixture was removed and 1 mL of complete medium with 10% FBS was added per well. The transfection effect was detected after a period of incubation of 48 h. Post transfection of PEI/ONs for duration of 5 h, HepG2-NTCP cells were inoculated with HBV at 1,000 genome equivalents (GE) per cell in a complete medium containing 2.5% DMSO, 4% PEG8000 for 12 h at 37°C and 5% CO_2_. Cell culture supernatants were harvested at 0, 4, and 8 days post-inoculation (dpi) for the measurement of HBsAg and HBeAg levels, which served as markers of infection.

### 2.4 Experiments in mice

The experiments were conducted using male specific pathogen-free BALB/c mice aged 6–8 weeks. For evaluating the therapeutic effects of PEI/ON in HBsAg, were hydrodynamiclly injected with pHBV1.3 plasmid through tail-vein at day 0. The 6-weeks-old BALB/c male mice (*n* = 6/group) were first injected with 15 μg of pHBV1.3 plasmid DNA on their tail veins through hydrodynamic injection (HDI), followed by injection of 300 uL PEI/ON complex solutions (12 uL PEI: 6 uL ON) which were administered every 5 days for a total of three doses. The mice were randomly divided into four groups and subjected to the injections of PBS, PEI, PEI/10 nt-AC complex and PEI/40 nt-AC complex, respectively. Serial serum samples and tissues were collected at the indicated time points or after sacrifice.

### 2.5 Cytotoxicity assay

An equal number of HepAD38 cells and HepG2-NTCP cells in 96-well plates were assayed after transfection. The cytotoxicity of PEI/oligonucleotide was measured by using the Cell Counting Kit-8 assay as per the manufacturer’s instructions (YiSheng biotech, Shanghai, China). Absorbance at 450 nm was measured using a multimode plate reader (Synergy 4, BioTek).

### 2.6 HBsAg and HBeAg quantification

Culture supernatants and cell lysates were collected to detect the secretion levels and the intracellular levels of HBsAg and HBeAg, respectively. HBsAg and Hepatitis B virus e antigen (HBeAg) levels were measured quantitatively using the Abbott Architect immunoassay system (Abbott Laboratories).

### 2.7 HBV DNA detection

HBV DNA was detected using the cell culture supernatant. Viral DNA analysis was then performed by using quantitative PCR (qPCR) kit and a 20 uL reaction volume (Tiangen Biotech, Beijing, China). Forward and reverse primers used were 5’-AATGCCCCTATCTTATCAACAC-3′ and 5′ -GAGATTGAGATCTTCTGCGACG-3′, respectively. Amplification was performed as follows: 95°C for 10 min, then 40 cycles of 95°C for 10 s, 55°C for 15 s, and 72°C for 20 s. Serial dilutions of a plasmid containing an HBV insert were used as quantification standards.

### 2.8 RNA isolation and northern blot hybridization

Total RNA purification was performed using the Trizol reagent according to the manufacturer’s instructions. 1ug of the total isolated RNA was subjected to treatment with 2.2 M formaldehyde, and subsequent electrophoresis using 1% agarose gel, after which bands were transferred onto a nylon membrane. The membrane-bound RNA was hybridized with a digoxigenin (DIG)-labeled HBV RNA probe specific for detection of genomic HBV RNA by using the DIG Northern starter kit (Roche Diagnostics), and the hybridization protocol was performed according to the manufacturer’s instructions. 1 μg of the purified RNA was subjected to electrophoresis on an agarose gel to detect 28S and 18S ribosomal RNAs (rRNAs) used as the loading control.

### 2.9 Detection of viral replicative intermediates and proteins

HBV core particles and HBV DNA replicative intermediates were extracted from the cytoplasmic fractions of cells and subjected to Southern blot analysis as per methods previously described (Lin et al., 2017). A DIG-labeled HBV RNA probe specific for detection of genomic HBV RNA was prepared using the DIG Northern starter kit (Roche Diagnostics), and the hybridization protocol was performed according to the manufacturer’s instructions. To calibrate the loading quantity for HBV replication, β-actin protein was determined by conducting sodium dodecylsulfate (SDS)-polyacrylamide gel electrophoresis and HBV core particles were resolved by performing native agarose gel electrophoresis using the same cellular lysates and were subjected to immunoblot analysis with antibodies to β-actin and HBcAg, respectively.

### 2.10 HBV production

For the production of HBV particles, HepAD38cells were cultured as per methods described above (Cells and reagents, Cell culture). Cells were maintained in the DMEM/F12 medium supplemented with 5% fetal bovine serum and 2% DMSO. HBV particles were concentrated from the purified HepAD38 supernatant by conducting overnight precipitation with 10% PEG 8000 and centrifugation at 4°C for 1 h at 16,000 *g*. The pellet was resuspended in the Opti-MEM medium supplemented with 2.5% DMSO at a concentration of 10^10^ GE per milliliter. HBV inoculum concentration was quantified using real-time PCR by following the protocol of HBV DNA detection.

### 2.11 Fluorescence microscopy

AD38 cells were seeded at a density of 1 × 10^6^ cells/well on circular glass coverslips placed in a 12-well plate and were grown overnight to obtain 70% confluence before subjection to transfection with FITC-labeled oligonucleotide or pEGPF-C2 for 5 h. For determination of transfection efficiency, green fluorescent protein (GFP) expression from the transfected plasmid pEGFP-C2 was monitored in living cells in culture using the Olympus CXC41 fluorescence microscope at a magnification of 100X. Cells were photographed at 48 h after transfection using bright-field and fluorescence microscopy.

For staining with anti-HBcAg antibodies after HBV infection, cells harvested at 8 days post injection were subjected to washing steps using PBS three times and fixation in 4% paraformaldehyde for 10 min at room temperature; thereafter, the cells were permeabilized with 0.2% Triton X-100 in PBS for 30 min. Detection of intracellular HBcAg levels was achieved after incubation with the anti-HBcAg antibody at 1:500 dilution overnight at 4°C. Coverslips were then washed twice with PBS and incubated with the secondary antibody for 1 h. Then coverslips were stained with 4′,6-diamidino-2-phenylindole (DAPI) and mounted on clean glass slides with a fluorescence-free glycerol-based medium and examined using a fluorescence microscope.

### 2.12 Immunohistochemically staining with anti-HBsAg and hematoxylin and eosin staining

Liver sections were embedded in paraffin and were then subjected to staining with anti-HBsAg or hematoxylin and eosin (H&E) to visualize the pattern of HBsAg expression and the inflammation status, respectively. The histological features of the tissues were observed and imaged using a light microscope (ECLIPSE 80i. Nikon Tokyo, Japan).

### 2.13 Statistical analysis

All data have been presented as mean ± standard deviation (SD), based on data calculated from at least three independent repeats. Statistical significance was examined using the Student’s *t*-test. A *p*-value <0.05 was considered statistically significant for all analyses. The GraphPad Prism 6 software was used for plotting data and for statistical analysis.

## 3 Results

### 3.1 Transfection of the oligonucleotides with PEI selectively induces a decline in the HBsAg levels in HepAD38 cells

**Figure 1:**
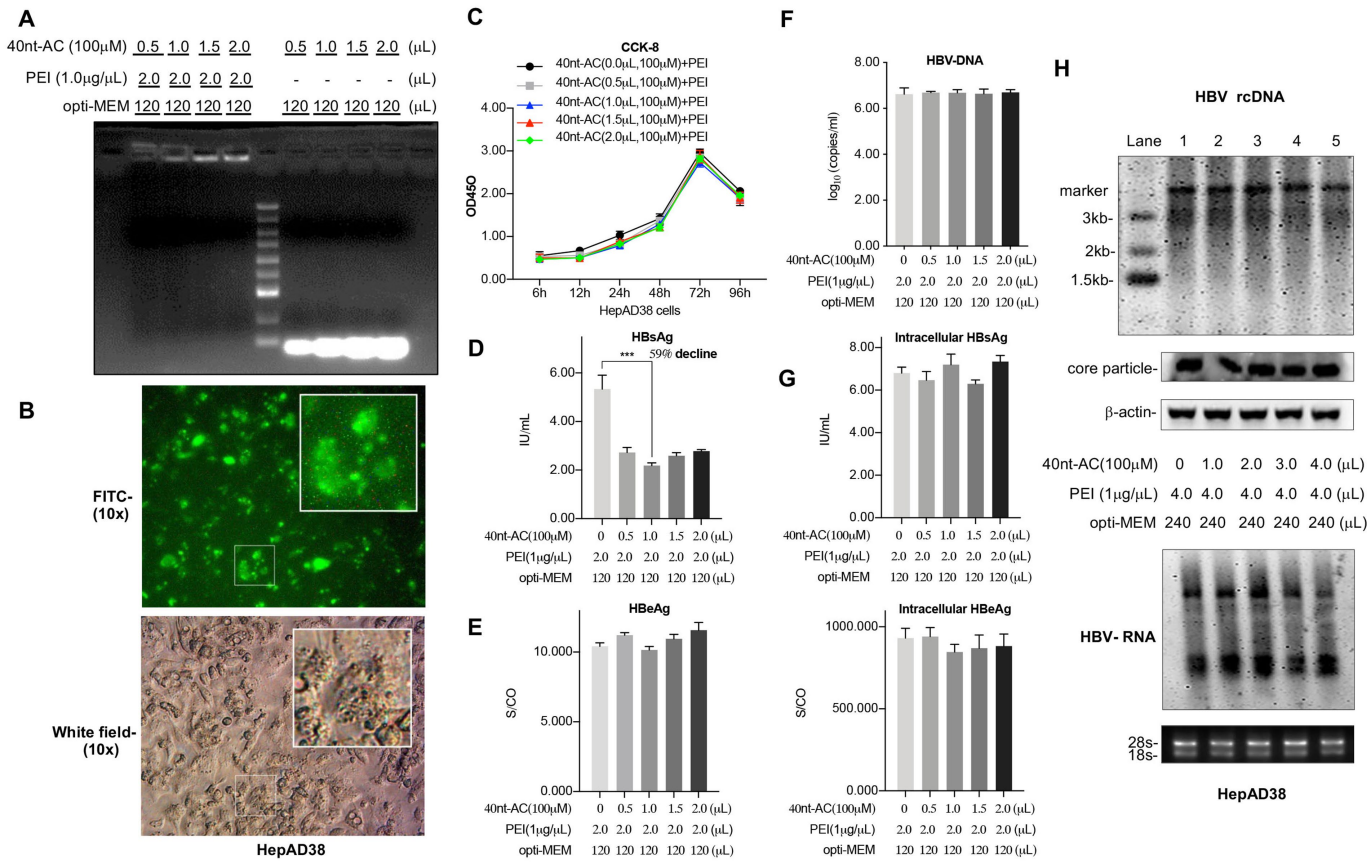
PEI/40 nt-AC complex selectively reduces the secretion of HBsAg in HepAD38 cells. **(A)** The gel retardation assay indicated that PEI complexing to 40 nt length oligonucleotide (adenosine-cytosine repeated sequences) at different weight ratio as indicated. 1 μL of 40 nt-AC water solution (100 μM) contains approximately 1.2 ug of 40 nt-AC oligonucleotide. **(B)** Internalization of FITC-labeled oligonucleotides in HepAD38 cells with fluorescent vision (up) and bright field (down). **(C)** The cytotoxicity of PEI/oligonucleotide was measured by using the Cell Counting Kit-8 assay. HepAD38 cells were cultured in 12-well plate and transfected with PEI/ON. The culture supernatants were collected to detect HBsAg **(D)**, HBeAg **(E)**, and HBV DNA **(F)** levels after 48 h. Means and SDs of data from three independent experiments are plotted. **(G)** HepAD38 cells were cultured in 6-well plate and transfected with PEI/ON as indicated. Cells were lysed with 100 μL of RIPA lysis buffer (25 mM Tris, pH 7.4, 150 mM NaCl, 1% NP-40, 1% sodium deoxycholate, 0.1% SDS). The intracellular levels of HBsAg and HBeAg were determined. **(H)** The replication intermediates in viral core particles were examined by Southern blot and Viral RNAs by Northern blot. RC, relaxed circular DNA. 18S/28S rRNAs served as the RNA loading control. Means and SDs of data from at least three independent tests were plotted. ***p < 0.001.

The previous study demonstrated that NAPs with a length of 40 nucleotides (poly-AC repeat-based sequence, 40 nt-AC) conferred the best antiviral effect against HBV (Guillot et al., 2017). Additionally, the heteropolymeric adenosine-cytosine sequences are appropriate because of their lack of secondary structure, antisense activity, or immunostimulatory activity (Guillot et al., 2017). To investigate the antiviral effect of the oligonucleotide (40 nt-AC) transfected with PEI, we used the human hepatoma cell line HepAD38 which harbored a replicating HBV genome for investigation. The complexation of PEI with 40 nt-AC was first detected by agarose gel electrophoresis (Figure 1A). This gel retardation assay indicated that the positive charge on PEI could neutralize the negative charge of the oligonucleotide and could thereby hinder the migration of oligonucleotides in the gel. Complete complexations of PEI and oligonucleotides at different ratios were successfully achieved in our experiment.

Besides, as shown in Figure 1B, via fluorescence microscopy, the FITC-labeled oligonucleotide (40 nt-AC) could be efficiently transfected with PEI into the AD38 cells. Furthermore, the cytotoxicity of PEI/ON complexes was determined to be non-toxic using the CCK-8 assay (Figure 1C) and MTT assay (Supplementary Figure 1) by comparing the cytoxicity of the complexes with the control, i.e., the PEI without an oligonucleotide.

The PEI/ON complex significantly reduced the HBsAg secretion levels in the supernatant by approximately 59% of the control level in a dose-independent manner. Approximately 2 uL of PEI (1 ug/uL) transfected with 1 uL of 100 uM of 40 nt-AC yielded the best inhibitory effect on the HBsAg level (Figure 1D). However, evident changes were not observed in the extracellular HBeAg level (Figure 1E) and the viral DNA (Figure 1F) and in the accumulation of intracellular HBsAg and HBeAg (Figure 1G). Additionally, HBV replication intermediates and mRNAs were further examined using Southern blot and Northern blot analyses, respectively. As illustrated in Figure 1H, we ruled out the possibility that PEI/ONs had an influence on the levels of 3.2 kb relaxed circular DNA (rcDNA), and 3.5 kb and 2.4 kb/2.1 kb transcripts of HBV. Transfection of PEI/ON selectively reduced the HBsAg level but did not reduce the HBeAg level and the DNA level. These findings revealed a selective inhibition by the PEI/ON complex in terms of the secretion levels of HBsAg.

### 3.2 PEI/pEGFP-C2 complexes exert no impact on the expression levels of HBsAg

**Figure 2:**
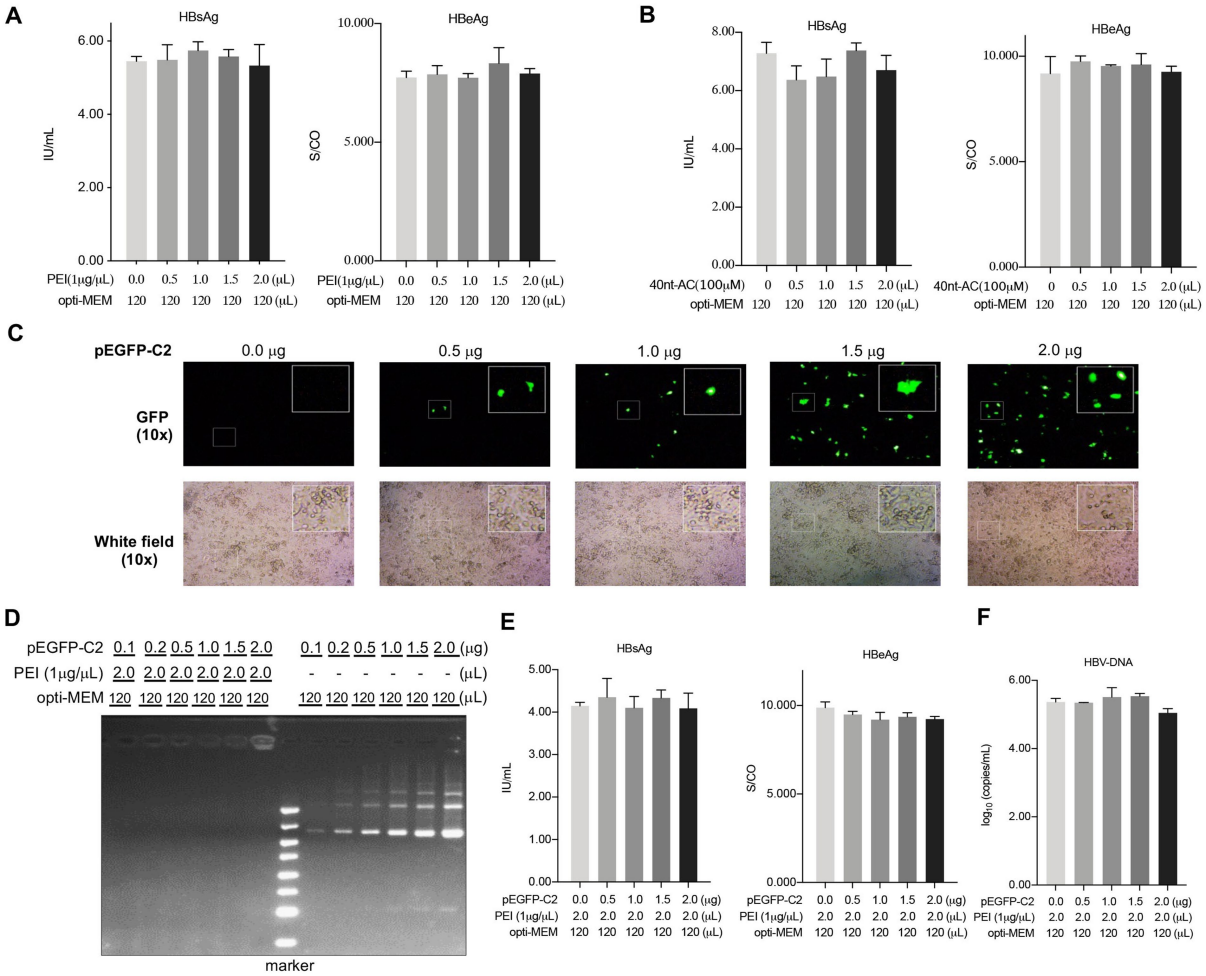
PEI/pEGFP-C2 complexes have no impact on the expression levels of HBsAg. HepAD38 cells were cultured in 12-well plate and treated with the PEI **(A)** or ON **(B)** alone and the culture supernatants were collected to detect HBsAg, HBeAg after 48 h. **(C)** Green fluorescent protein (GFP) expression in cells in a dose dependent manner with fluorescent vision (up) and bright field (down) by fluorescence microscope. **(D)** The gel retardation assay indicated that PEI complexing to pEGFP-C2 at different weight ratio as indicated. The culture supernatants were collected to detect HBsAg, HBeAg **(E)** and HBV DNA level **(F)** after 48 h of transfection. Means and SDs of data from three independent experiments are plotted.

To exclude the possibility that free PEI or oligonucleotides alone in cells could influence the activities of HBV, HepAD38 cells were subjected to separate treatments with PEI or oligonucleotides alone in a dose-dependent manner. Results showed that there were no apparent changes in the levels of HBsAg and HBeAg in the culture supernatants subjected to treatment with either PEI (Figure 2A) or oligonucleotides (Figure 2B). These data emphasized the importance of the formation of the complex of PEI and oligonucleotide for the exhibition of inhibited activity.

Considering that the delivery of short oligonucleotides using PEI inhibited HBsAg secretion in HepAD38 cells (Figure 1), we aimed to explore whether PEI/plasmid-DNA complexes might also exert its antiviral activity in HBV. To test this hypothesis, the plasmid pEGFP-C2 (4.7 kilo base pair-long) that encoded an enhanced green fluorescent protein (EGFP) under the influence of the cytomegalovirus promoter was transfected with PEI into the HepAD38 cells in a dose-dependent manner (Figure 2C). The gel retardation assay was used to comfirm complexation of PEI with pEGFP-C2 at different weight ratios as depicted in Figure 2D. Importantly, the levels of secreted HBsAg and HBeAg (Figure 2E) and HBV-DNA (Figure 2F) were not affected by the PEI/plasmid-DNA transfection. This suggested that the formation of the PEI/ON complex might demonstrate different physicochemical properties that play a specific role in its inhibited activity.

### 3.3 HBsAg level is suppressed by PEI/ON complexes in an oligonucleotide length-dependent manner

**Figure 3:**
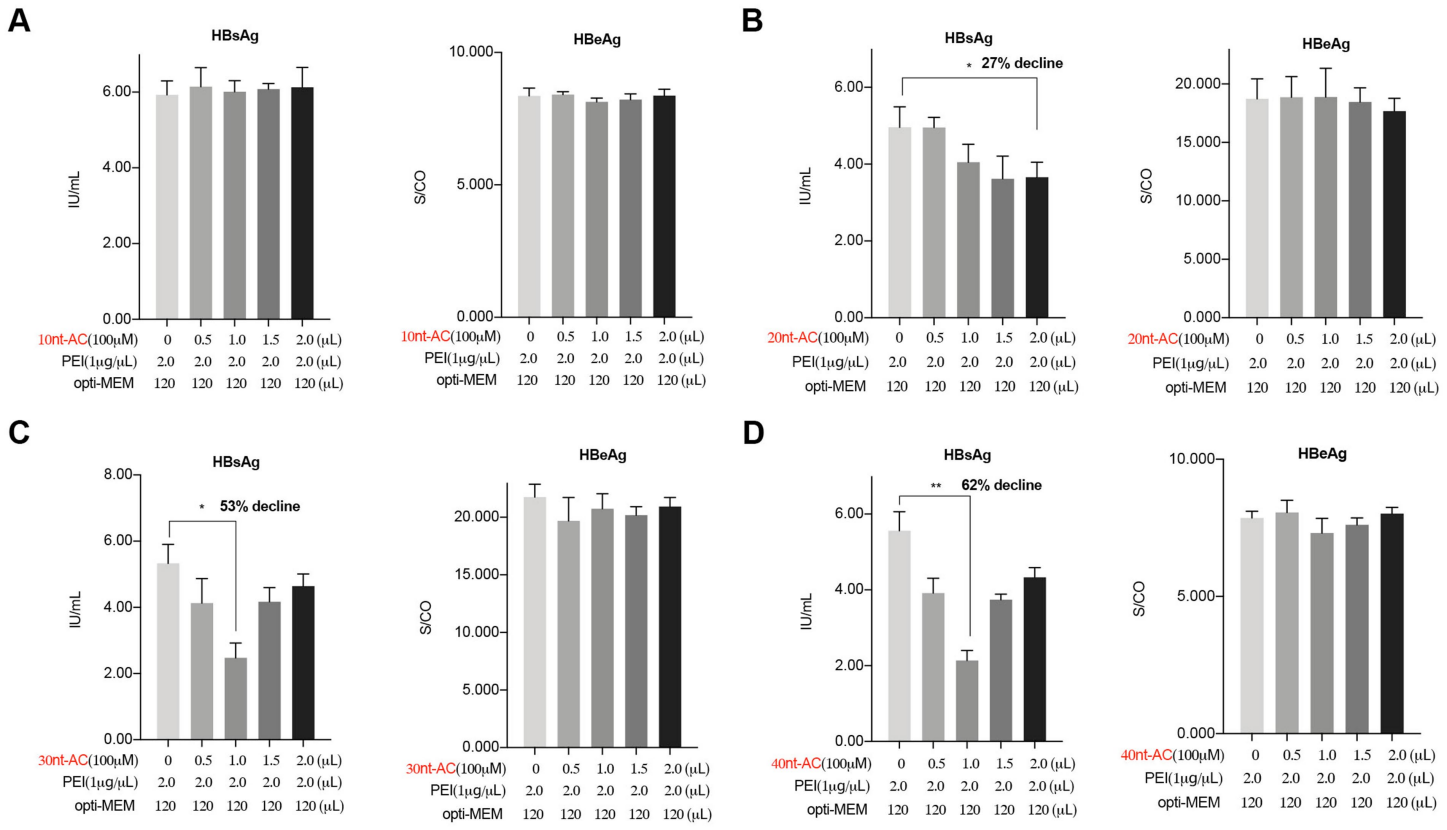
PEI/ON complexes inhibit secreted HBsAg in a size dependent manner. HepAD38 cells cultured in 12-well plate were transfected with unmodified oligodeoxynucleotides (100 μM) in different indicated nt-length using PEI (1 μg/μL). **(A)** The oligodeoxynucleotide in 10 nt length, 10 nt-AC. **(B)** The oligodeoxynucleotide in 20 nt length, 20 nt-AC. **(C)** The oligodeoxynucleotide in 30 nt length, 30 nt-AC. **(D)** The oligodeoxynucleotide in 40 nt length, 40 nt-AC. Post 48 h transfection, the culture supernatants were collected to detect HBsAg, HBeAg. Means and SDs of data from three independent experiments are plotted. *p < 0.05, **p < 0.01.

To assess the oligodeoxynucleotide size effects on inhibiting activity, four types of AC-repeated oligonucleotides with different lengths (10, 20, 30, and 40 nucleotides) were transfected using PEI into the HepAD38 cells. The results showed that the suppression of the secreted HBsAg was lost in the 10-nt oligonucleotide (Figure 3A), while the secretion of HBsAg decreased gradually in the 20-nt oligonucleotide in a dose-dependent manner (Figure 3B). Interestingly, the treatment of the 30-nt and the 40-nt oligonucleotides with PEI resulted in a strong inhibitory activity, with a 53% (Figure 3C) and a 62% reduction (Figure 3D) in the HBsAg levels in a dose-independent manner. Meanwhile, these four nucleotides with PEI exerted no effects on the HBeAg level as shown in Figure 3. These results indicated that a length of 40 nucleotides was optimal for the efficient inhibition of HBsAg secretion by PEI/ONs.

### 3.4 PEI/ON complexes uniquely inhibit HBsAg secretion compared to other commercial transfection reagents

**Figure 4:**
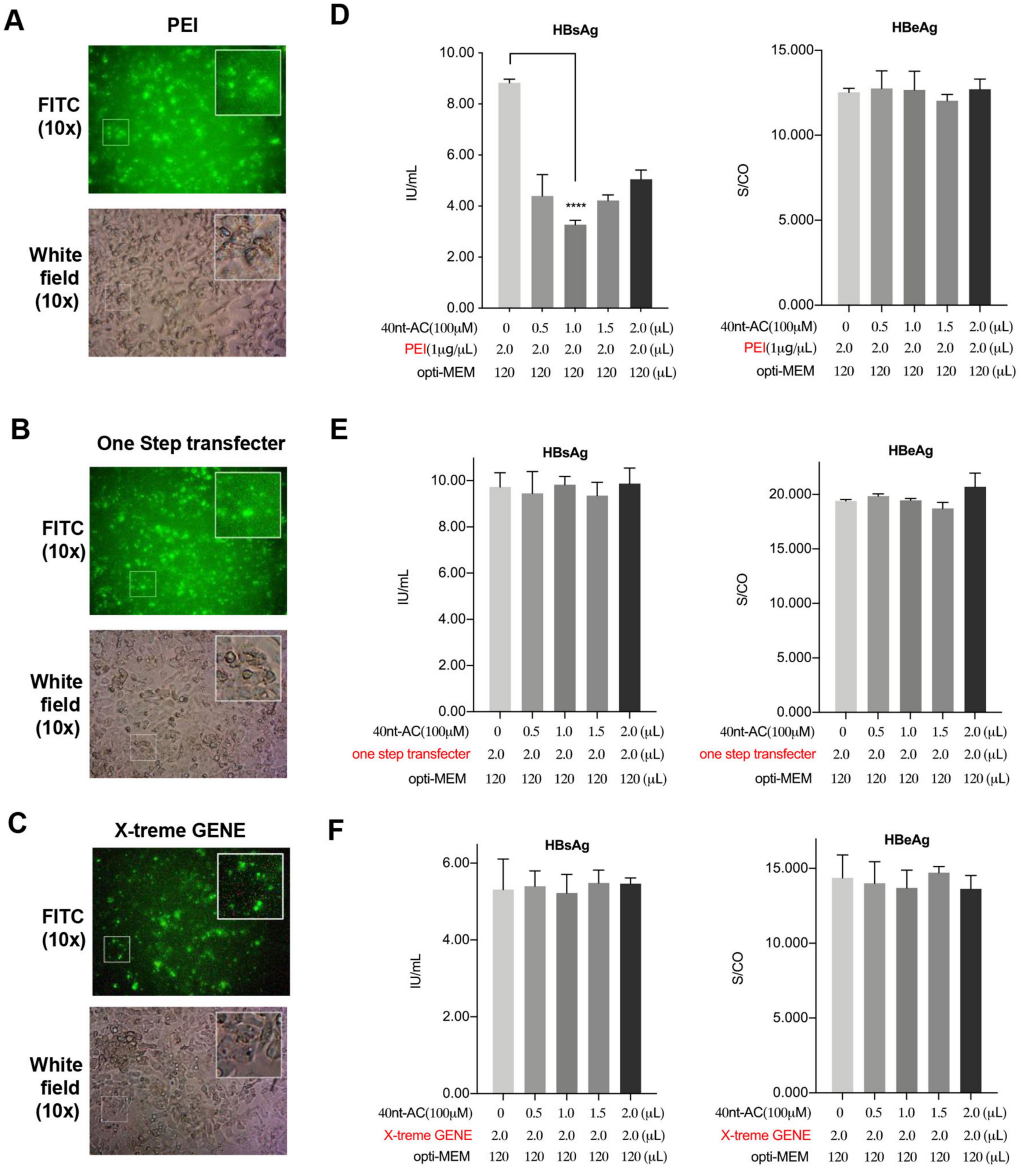
PEI/ON complexes uniquely inhibit HBsAg secretion comparing with other commercial transfection reagents. HepAD38 cells cultured in 12-well plate were transfected with 40 nt-AC oligonucleotides using the indicated commercial transfection reagents. 48 h post of transfection, internalization of FITC-labeled oligonucleotides in HepAD38 cells in fluorescent vision (up) and white field (down) by using different commercial transfection reagents. **(A)** PEI. **(B)** OneStep transfecter: One-step DNA Transfecter (ENLIGHTEN Biotech, LD210-005). **(C)** X-tremeGENE: X-tremeGENE 9 DNA Transfection Reagent (Roche, 06365779001). **(D–F)** The culture supernatants were collected to detect HBsAg and HBeAg levels after 48 h of transfection. ****p < 0.0001.

To detect whether other commercial transfection reagents could replace PEI to deliver the oligonucleotides that would result in an inhibited activity in HBsAg level, we confirmed that the successful internalization of the FITC-oligonucleotide (40 nt-length, poly AC repeat-based) were loaded with PEI (Figure 4A), OneStep-transfecter (Figure 4B) and X-tremeGENE (Figure 4C) in HepAD38 cells, respectively. The cell culture supernatant was collected for detection. Interestingly, results indicated that only PEI that undergo complexation with oligonucleotides could reduce the levels of secreted HBsAg without a decline in the HBeAg level (Figure 4D). However, oligonucleotides that were harbored by the other two commercial transfection reagents exerted no effects on HBsAg or HBeAg levels as shown in Figure 4E (OneStep-transfecter) and Figure 4F (X-tremeGENE). We further performed transfection of different length oligonucleotides (10 nt, 20 nt, 30 nt and 40 nt) with two commercial kits (OneStep-transfecter and X-tremeGENE) in HepAD38 cells. As shown in Supplementary Figure 2, no apparent changes were observed in the levels of HBsAg and HBeAg. These results demonstrated that PEI and oligonucleotides at a certain length were indispensable to the formation of functional complexes that inhibited HBsAg secretion.

### 3.5 The complex of PEI with a decoy oligonucleotide inhibits HBsAg secretion but does not decrease HBV transcripts

**Figure 5:**
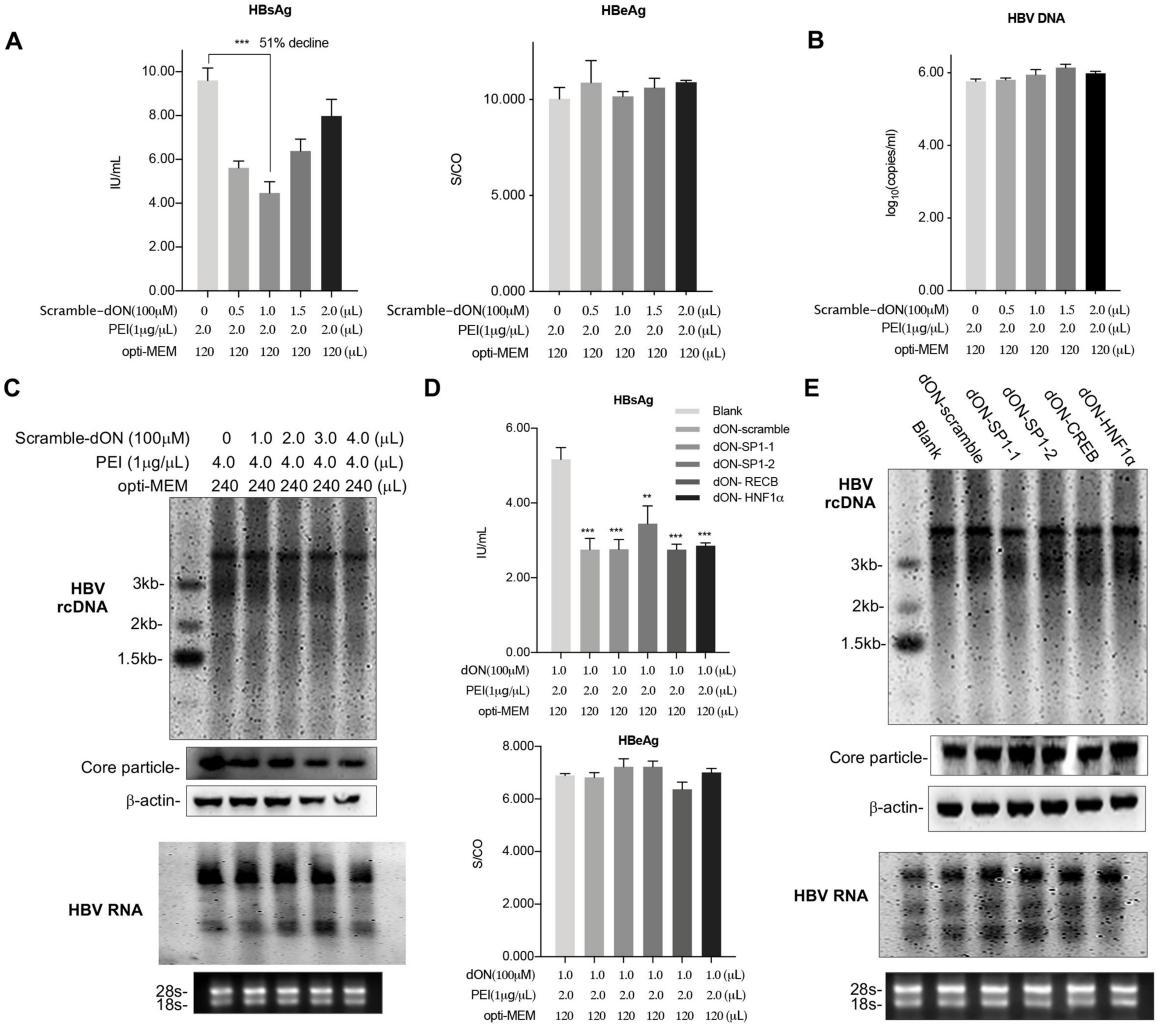
PEI/Decoy-ON inhibits HBsAg secretion levels but not HBV transcripts. HepAD38 cells cultured in 12-well plate were co-transfected with scramble oligonucleotides with different initial weight ratios (PEI: ON) as indicated. The culture supernatants were collected to detect HBsAg and HBeAg levels **(A)** and HBV DNA levels **(B)** after 48 h of transfection. **(C)** HepAD38 cells were cultured in 6-well plate and transfected with scramble ON using PEI as indicated. HBV replication intermediates in viral core particles were examined with Southern blot and Viral RNAs with Northern blot. **(D)** The HBsAg levels (up) and HBeAg levels (down) in supernatants of cells transfected with the scramble double-stranded oligonucleotide or decoy oligonucleotides of Sp1, CREB and HNF1α, respectively. Sp1, Specificity protein 1; HNF1α, hepatocyte nuclear factor 1α; CREB, C-AMP-response element binding protein. **(E)** HepAD38 cells cultured in 6-well plate were transfected with PEI/decoy-ON as indicated. HBV replication intermediates in viral core particles were examined with Southern blot and Viral RNAs with Northern blot. rcDNA, relaxed circular DNA. 18S/28S RNAs served as the RNA loading control. Means and SDs of data from three independent experiments are plotted. **p < 0.01, ***p < 0.001.

The decoy oligonucleotide (decoy-ON, dON) is short, double-stranded DNA molecules that interfered with gene expression at the transcription level through the simulation of a binding ligand with their target transcriptional factor (Billioud et al., 2016). We also explored whether PEI complexes with double-stranded oligonucleotides could also inhibit HBsAg secretion. To this end, we employed a scramble decoy-oligonucleotide, which formed complexes with PEI to transfect HepAD38 cells in a dose-dependent manner. Similar to the results obtained for single-stranded oligonucleotides, PEI complexation with double-stranded oligonucleotides reduced HBsAg levels (Figure 5A). The levels of HBeAg (Figure 5A, right panel) and HBV DNA (Figure 5B) also demonstrated no alterations. Concurrently, Southern blot and Northern blot results showed that the expected changes in the levels of both viral rcDNA and mRNAs did not decrease upon PEI transfection of HepAD38 cell (Figure 5C).

To ascertain whether PEI/decoy-ON inhibited HBV gene expression and replication, five decoy-oligonucleotides for the Specificity protein 1 (*Sp1*), (Raney et al., 1992) hepatocyte nuclear factor 1α (*HNF1α*), (Raney et al., 1991) and C-AMP-response element binding protein (*CREB*), (Kim et al., 2008) which were key regulators for HBV replication and transfection, and scramble as a control group were included. All PEI/decoy-ONs transfected could reduce HBsAg levels in the supernatant, but the levels of HBeAg demonstrated no changes (Figure 5D). We further determined the effect of PEI/decoy-ON transfection on HBV DNA replication by performing Southern blot hybridization for the isolated DNA samples derived from core particles and ascertained HBV RNA levels by performing Northern blot detection. Treatment of cells with a set of decoy-ONs for HBV relative transcriptional factor did not reduce HBV replication and transcription compared to scramble-decoy-oligonucleotide (Figure 5E). In summary, decoy-ON as double strand oligonucleotides complexing with PEI showed remarkable reduction of HBsAg levels in a sequence-independent manner, which was similar to the PEI/ON complexes.

### 3.6 Treatment of PEI/oligonucleotide prior to viral inoculation inhibits HBV infection

**Figure 6:**
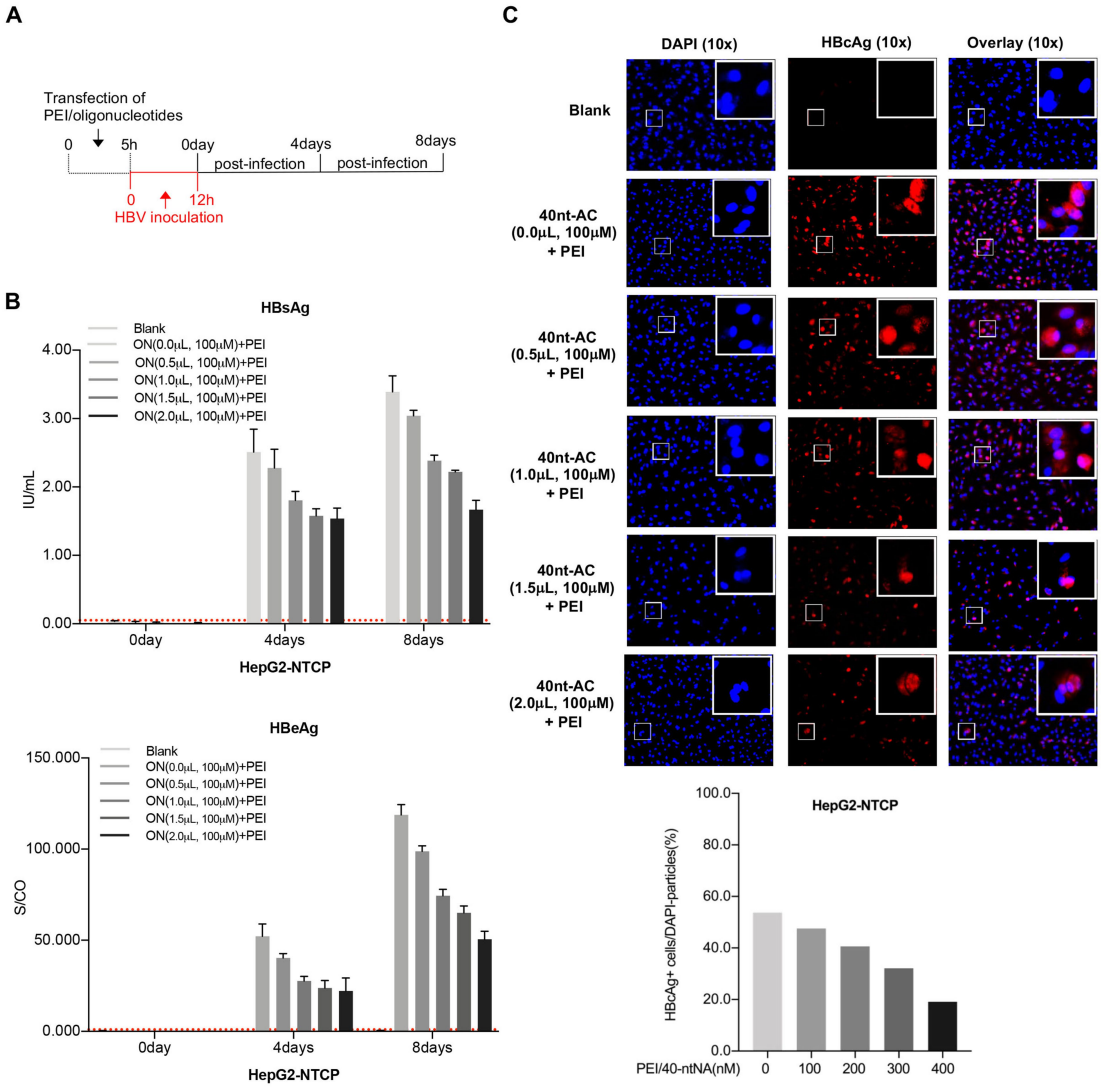
PEI/oligonucleotide transfection prior to viral inoculation inhibits HBV infection in HepG2-NTCP cells. **(A)** Treatment procedure: HepG2-NTCP cells were transfected by the indicated ratios of PEI/ONs for 5 h, following by 12 h of inoculation with HBV at 1,000 GE per cell. Supernatants were harvested for secreted HBsAg, HBeAg in day 0, 4, and 8 post-inoculation. **(B)** The culture supernatants were collected to detect both secreted HBsAg and HBeAg at day 0, 4 and 8 post-infection. Dotted lines represent cut-off thresholds. **(C)** Post 8 day infection, HBcAg were detected by anti-HBc with fluorescent tag (red) in cells in which nuclei were labeled with DAPI (blue) by fluorescence microscopy. The exact numbers of DPAI dying and HBcAg (+) cells were analyzed and summarized by Image J software. All data, expressed as means ±standard deviation, were independently reproduced two times.

For establishing HBV infection, the supernatant from HepAD38 cells was used as an inoculum. To assess the possible effect of the complex on the natural HBV infection, HepG2-NTCP cells were transfected with PEI/ON complexes in different ratios for 5 h. They were then inoculated with HBV at 1000 GE per cell for 12 h. HBV infection was assessed by measuring the HBsAg and HBeAg levels in supernatants at 4 and 8 dpi (Figure 6A). As shown in Figure 6B, PEI/ON transfection prior to HBV infection resulted in significant reductions of HBsAg and HBeAg secretion in a dose-dependent manner. At day 8 post-inoculation, cells were harvested and analyzed for the presence of HBcAg using fluorescence microscopy (Figure 6C). A dose-dependent inhibition of the HBV infection was observed under different concentrations of oligonucleotides with PEI, thus demonstrating that PEI/ON complexes were indeed active during viral infection (Figures 6B,C). Additionally, the count of HBcAg (+) cells (HBV-infected cells) enabled a more accurate assessment of the inhibition of PEI/ONs on viral entry in our experiments (Figure 6C, bottom). As shown in Supplementary Figure 3, PEI/ON transfection did not exert obvious cytotoxic effect by comparing with the blank group.

### 3.7 PEI/40nt-AC complex reduces HBsAg levels in the BALB/c mice hydrodynamically injected with the HBV-expressing plasmid

**Figure 7:**
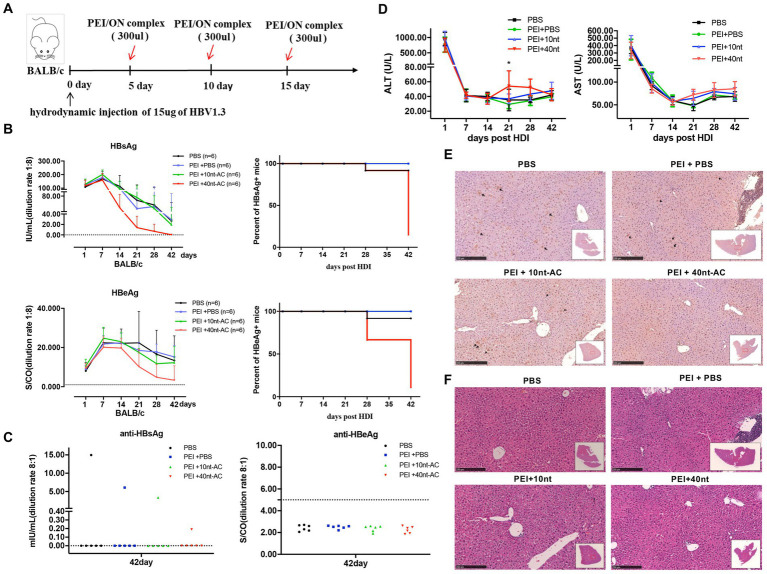
PEI/40 nt-AC complex reduces HBsAg level in the HDI BALB/c mice. **(A)** Schematic representation of the mice experiment. The 6-weeks-old BALB/c male mice (*n* = 6/group) were hydrodynamiclly injected with pHBV1.3 plasmid through tail-vein at day 0. The injections of PBS, PEI, PEI/10 nt-AC complex and PEI/40 nt-AC complex are indicated by red arrows. **(B)** Serum samples were collected at indicated time points and the levels of serum HBsAg and HBeAg kinetics in mice were analyzed. Cut-off thresholds of determination are 0.05 IU/mL for HBsAg and 1.000 S/CO for HBeAg, which are represented by dotted lines. The positive rates of serum HBsAg and HBeAg in mice were analyzed and presented (right panel). **(C)** Sera were collected at 42 days post of pHBV1.3-injection and the levels of anti-HBsAg (left) and anti-HBeAg (right) were analyzed. **(D)** Serum levels of alanine transaminase (ALT) and aspartate transaminase (AST) were measured. A two-tailed Student t test was used. *p < 0.05. **(E)** Mice were sacrificed at 42 days post of pHBV1.3 injection. Liver sections taken from HDI-mice in different groups were stained for HBsAg (black arrow heads) in addition to **(F)** HE staining. Representative images from control and PEI/ON transfected mice are shown. Scale bar = 250 μm.

To further evaluate the anti-HBsAg efficacy of PEI/ON *in vivo*, we utilized BALB/c mice (*n* = 6/group) that were hydrodynamically injected with pHBV1.3 (15 ug/mouse) as the testing model. The experimental design is illustrated in Figure 7A. Approximately 300 uL of the PEI/ON complex solutions were injected through the tail vein and were administered every five days for a total of three doses after performing the pHBV1.3 injection at day 0. Sera were collected at the time points indicated for the analysis of HBsAg, HBeAg, anti-HBsAg, and anti-HBeAg levels. The results revealed that serum HBsAg and HBeAg levels were both significantly reduced by the injection of PEI/40 nt-AC, compared with PBS, PEI/PBS, and PEI/10 nt-AC groups at day 42 (Figure 7B). A total of five mice in the PEI/40 nt-AC group (*n* = 6) and 1 mouse in the PBS group (*n* = 6) presented with HBsAg and HBeAg losses in serum at day 42 (Figure 7B, right panels). However, only 1 mouse in the PEI/40 nt-AC group (*n* = 6) was anti-HBsAg-positive (Figure 7C, left panel), while anti-HBeAg-positive serum at day 42 (Figure 7C, right panel) was not detected in any mouse. Furthermore, the serum levels of aspartate aminotransferase (AST) and alanine aminotransferase (ALT) were measured to determine the degree of liver damage. As shown in Figure 7D, transient ALT elevations peaked at days 21 and 28 but reverted to normal levels at day 42. Additionally, liver tissues were obtained from each group of mice who were sacrificed at day 42 and subjected to immunohistochemistry and hematoxylin and eosin (HE) staining to detect HBsAg levels and liver damage. The results demonstrated that HBsAg-positive cells were not detected in mice injected with PEI/40 nt-AC (Figure 7E). There were no remarkable hepatocyte cellular changes observed in the liver section of the four groups (Figure 7F; Supplementary Figure 4). To further confirmed the specificity of the inhibition of PEI/ON complex in secreted HBsAg level, plasmid pNL-sNLuc which can express secreted form of Nano-Luciferase (sNLuc) was employed and delivered into BALB/c mice via hydrodianic injection (HDI). As shown in Supplementary Figure 5, the sNLuc level in mouse sera sample did not impaired by the transfection of PEI/40 nt-AC complex, which was different with the observation in pHBV1.3-mice and confirmed that PEI/40 nt-AC transfection had no impact on plasmid expression in mouse model and exerted its specific inhibiting in HBsAg secretion.

In summary, these data suggested that PEI/40 nt-AC induced HBsAg level decline and promoted HBV clearance independent of the anti-HBsAg development. However, these changes were associated with a transient elevation of ALT, which reflected liver inflammation.

## 4 Discussion

Oligonucleotide, as a membrane impermeable molecule, cannot easily gain entry into the target intracellular activity sites (Shahbazi et al., 2016). Branched and linear PEI are promising candidate non-viral vectors for plasmid and oligonucleotide delivery both *in vitro* and *in vivo*, due to their remarkable transfection efficiencies with high complex stability. The preparation of PEI/ON complex is also easy and inexpensive (Pandey and Sawant, 2016; Yan et al., 2014; Patnaik and Gupta, 2013).

In this study, we made two transfections in HepG2 cells, i.e., 24 h after the first transfection of pHBV1.3 the second transfection with PEI/ON was carried out. The detected results showed that PEI/ON could effectively reduce HBsAg and HBeAg antigen expression under the two-transfection condition in a 40 nt oligonucleotide dose-independent manner (Supplementary Figure 6). It seems that the reduction of both HBsAg and HBeAg were due to the total suppression of HBV gene expression. However, pHBV1.3 gene expression required time to accumulate a measurable difference and we also can not completely rule out the possibility that the second transfection itself may interfere with the pHBV1.3 gene expression or aggravate cytotoxicity. Therefore, to further confirm the data obtained, HepAD38 cell which harbored a replicating HBV genome employed was employed. By comparing with the other two commercially available transfection reagents, PEI transfection with unmodified oligonucleotides showed a unique ability to inhibit HBsAg secretion without any accumulation of intracellular HBsAg. Meanwhile, there were no marked activities against HBeAg and HBV-DNA replication. These observations implied that PEI/oligonucleotide complexes were activated against the release of HBV SVPs, which were similar to the data obtained using the HepG2.2.15 cells and the duck Hepatitis B virus (DHBV) models upon treatment with NAPs, and this activation was driven specifically by the presence of the phosphorothioate modification in oligonucleotide to exert antiviral effects (Blanchet et al., 2019; Noordeen et al., 2013). However, the inhibition mechanisms involved in the PEI/ON complex were likely to be significantly different with NAPs in our study. Considering that treatment of HepAD38 cells with free PEI did not impair HBsAg levels, the use of PEI/ON as a complex was a prerequisite for the specific inhibition of secreted HBsAg. Notably, unmodified phosphodiester oligonucleotides exhibited no inherent ability for blocking viral entry or for exertion of post-entry effects in HBV.

It was reported that the plasmid DNA was over several thousand base pairs in length and harbored a myriad negative charges, while oligonucleotide such as siRNA was 20 base pairs in length and harbored a charge of only −40 (Ziebarth et al., 2017). The PEI/ON complex formed by electrostatic interaction was less stable than the PEI/pDNA complex and dissociated more efficiently to release the oligonucleotide and PEI into the cytoplasm. However, the mechanism targeted by the PEI/ON complex that underlies the post entry anti-HBsAg effect remained unclear. We partially speculated that the non-specific role of PEI in displacing the lipid might affect lipid metabolism involved in the assembly and secretion of SVP (Bertschinger et al., 2006; Hong et al., 2006; Gavilanes et al., 1982). This hypothesis was also supported by experiments using other transfection agents that provided similar intracellular oligonucleotide delivery, which lacked the inhibition activity in HBsAg levels (Figure 4). This was worthy of further investigation.

The transfection of PEI/ON complexes resulted in the reduction of secretion of HBsAg in an oligonucleotide size-dependent manner. As shown in Figure 3, the transfection of PEI/40 nt-AC complexes, and not PEI/10 nt-AC at the same treatment concentration, inhibited HBsAg secretion levels in HepAD38 cell supernatants. 1 uL of 40 nt-AC water solution (100 uM) contains approximately 1.2 ug of 40 nt-AC oligonucleotide. The mixture of DNA and transfection regent at 1 to 2 weight ratio has best transfection efficiency according to the manufacturer’s recommendations. A potential explanation was that PEI that was bound to oligonucleotides with different lengths or in different concentrations could form different types of polymers with different chemical properties. The complexes of oligonucleotides (greater than 20 nt in length or in certain concentration) binding to PEI (but markedly smaller than the PEI/pDNA complex size) with specific physicochemical properties might demonstrate a higher transfection efficiency and participate in the assembly of SVPs. Furthermore, the formation of PEI/ON complexes in a certain weight ratio exhibited higher transfection efficiency and appropriate stability and demonstrated an optimal anti-HBsAg activity, which also revealed the best balance between transfection efficiency and complex stability of PEI/ONs.

In the present study, we showed that the transfection of PEI/oligonucleotide prior to viral inoculation inhibited HBV infection in a dose dependent manner, which could be explained by the fact that free PEI or PEI/ON complex blocked HBV entry by competing with cell heparan sulfate proteoglycan to establish interaction with the low-affinity HBV envelope proteins, thereby preventing viral attachment to hepatocytes (Ruponen et al., 1999; Leistner et al., 2008). There was another reason that the PEI proton sponge effect that induced the rupture of the endosome/lysosome could have interfered with the pathways involved in HBV entry (Pandey and Sawant, 2016; Herrscher et al., 2020). Additionally, the most important observation was that PEI/ON complex transfection mediated the suppression of HBsAg synthesis or its secretion into the blood, thereby resulting in the clearance of HBV and reduction of HBV persistence in mice. This was consistent with previous observations that HBV clearance was not associated with hepatitis B surface antibody (HBsAb) development but coincided with transient ALT elevations, which might be related to the CD8 + T cell immune control (Shen et al., 2017; Shen et al., 2020).

We further confirmed that transfection of PEI/decoy-oligonucleotides including scramble oligonucleotide also resulted in the decline of HBsAg levels in HepAD38 cell cultures, which suggested that its anti-HBsAg effect is in a sequence-independent manner in our experiments. However, the transfection of PEI/decoy-oligonucleotide failed to show inhibitory activities in the HBV transcription, which was more likely that the sequences of decoy oligonucleotide were not designed efficiently. It was also possible that the results of rapid degradation of the unmodified oligonucleotide by nuclease (Spitzer and Eckstein, 1988) or the compensations by several other transcription factors were involved in regulation of HBV transcription when decoy-oligonucleotides were applied to HBV genome integrating-cells. Since siRNA, a double-stranded oligonucleotide showed remarkable ability to directly disrupt HBsAg mRNA levels, (Sioud, 2015), the use of PEI/siRNA complexes to reduce HBsAg and HBV mRNA levels will be an efficient strategy in our future exploration.

## 5 Conclusion

The present study was the first to investigate and elucidate that transfection of PEI complexes with oligonucleotides is a potential therapeutic method in decreasing HBsAg secretion compared with the use of other commercial transfection reagents. Although the PEI/oligonucleotide complexes did not exert significant cytotoxicity within a certain concentration range used in our experiments, the limited clinical potential for PEI-based therapies was attributed to cytotoxicity. More modifications of the PEI/ON complex are warranted to improve its transfection efficiency and to reduce its cytotoxicity before a real-world application in clinical settings.

## Data Availability

The original contributions presented in the study are included in the article/Supplementary material, further inquiries can be directed to the corresponding author.

## Glossary

AST: Aspartate aminotransferase
ALT: Alanine aminotransferase
CMV: Cytomegalovirus
CCK-8: Cell Counting Kit-8
CREB: C-AMP-response element binding protein
DMEM: Dulbecco’s modified Eagle’s medium
dpi: Days post-injection
DIG: Digoxigenin
DAPI: 4′,6-diamidino-2-phenylindole
dON: Decoy oligonucleotide
DHBV: Duck hepatitis B virus
EGFP: Enhanced green fluorescent protein
FBS: Fetal bovine serum
FITC: Fluorescein isothiocyanate
GFP: Green fluorescent protein
HBV: Hepatitis B virus
HBsAg: HBV surface antigen
HBcAg: Hepatitis B virus core antigen
HDI: Hydrodynamic injection
HBeAg: Hepatitis B virus e antigen
HNF1α: Hepatocyte nuclear factor 1α
H&E: Hematoxylin and eosin
HBsAb: Hepatitis B surface antibody
NAP: Nucleic acid polymer
NTCP: Sodium/taurocholate cotransporting polypeptide
ON: Oligonucleotide
PEI: Polyethylenimine
qPCR: Quantitative polymerase chain reaction
rRNA: Ribosomal RNA
rcDNA: Relaxed circular DNA
SVP: Subviral particle
siRNA: Small interference RNA
SDS: Sodium dodecylsulfate
SD: Standard deviation
Sp1: Specificity protein 1
