# Supplementary material for: Transfection of unmodified oligodeoxynucleotide with polyethylenimine reduces the level of hepatitis B surface antigen
Source: Front Microbiol. 2025 May 1;16:1600679. doi: 10.3389/fmicb.2025.1600679 (PMC12078216; doi:10.3389/fmicb.2025.1600679)
Supplement: Supplementary file 1 [file Image_1.pdf]

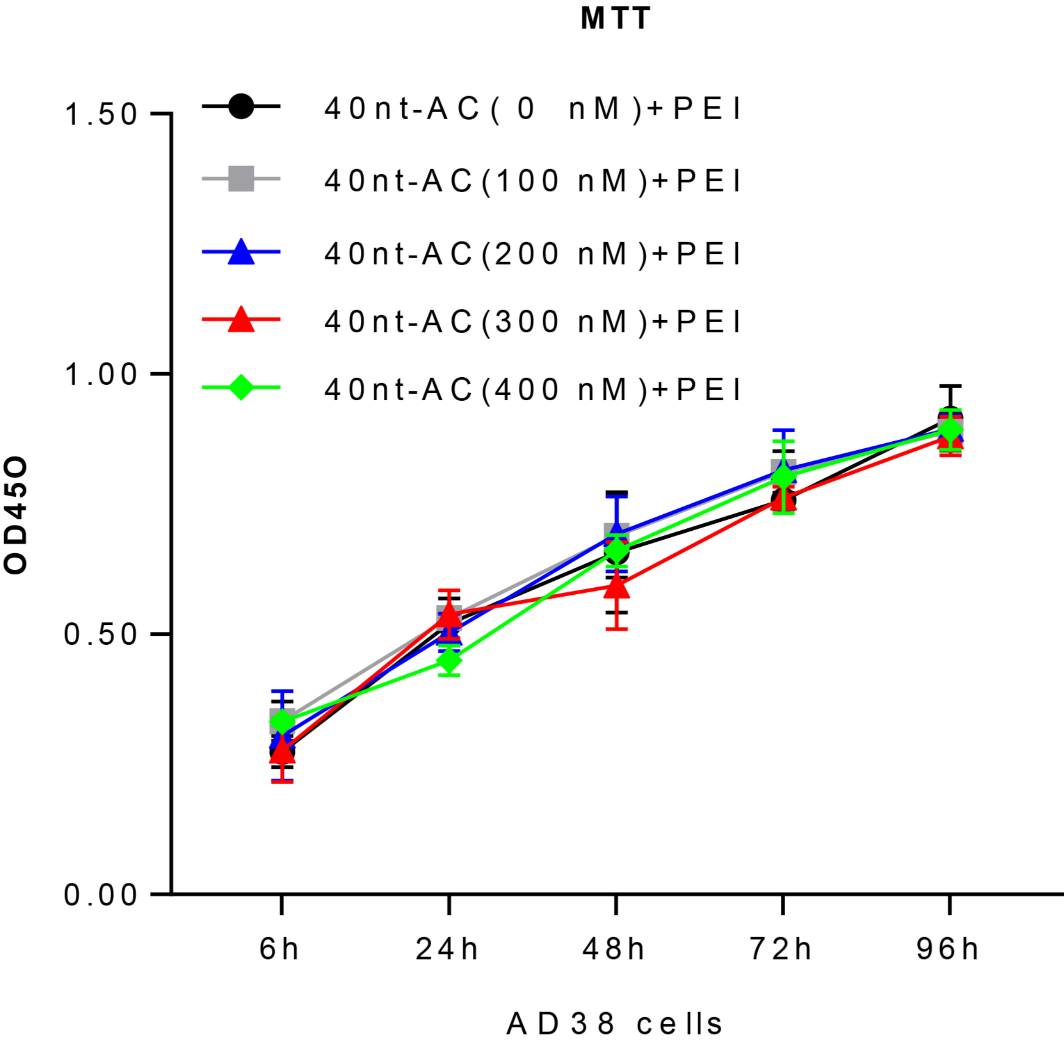

Supplementary Figure 1. The cytotoxicity of PEI/oligonucleotide was measured by using the MTT cell proliferation assay.
