# Supplementary material for: Transfection of unmodified oligodeoxynucleotide with polyethylenimine reduces the level of hepatitis B surface antigen
Source: Front Microbiol. 2025 May 1;16:1600679. doi: 10.3389/fmicb.2025.1600679 (PMC12078216; doi:10.3389/fmicb.2025.1600679)
Supplement: Supplementary file 2 [file Image_2.pdf]

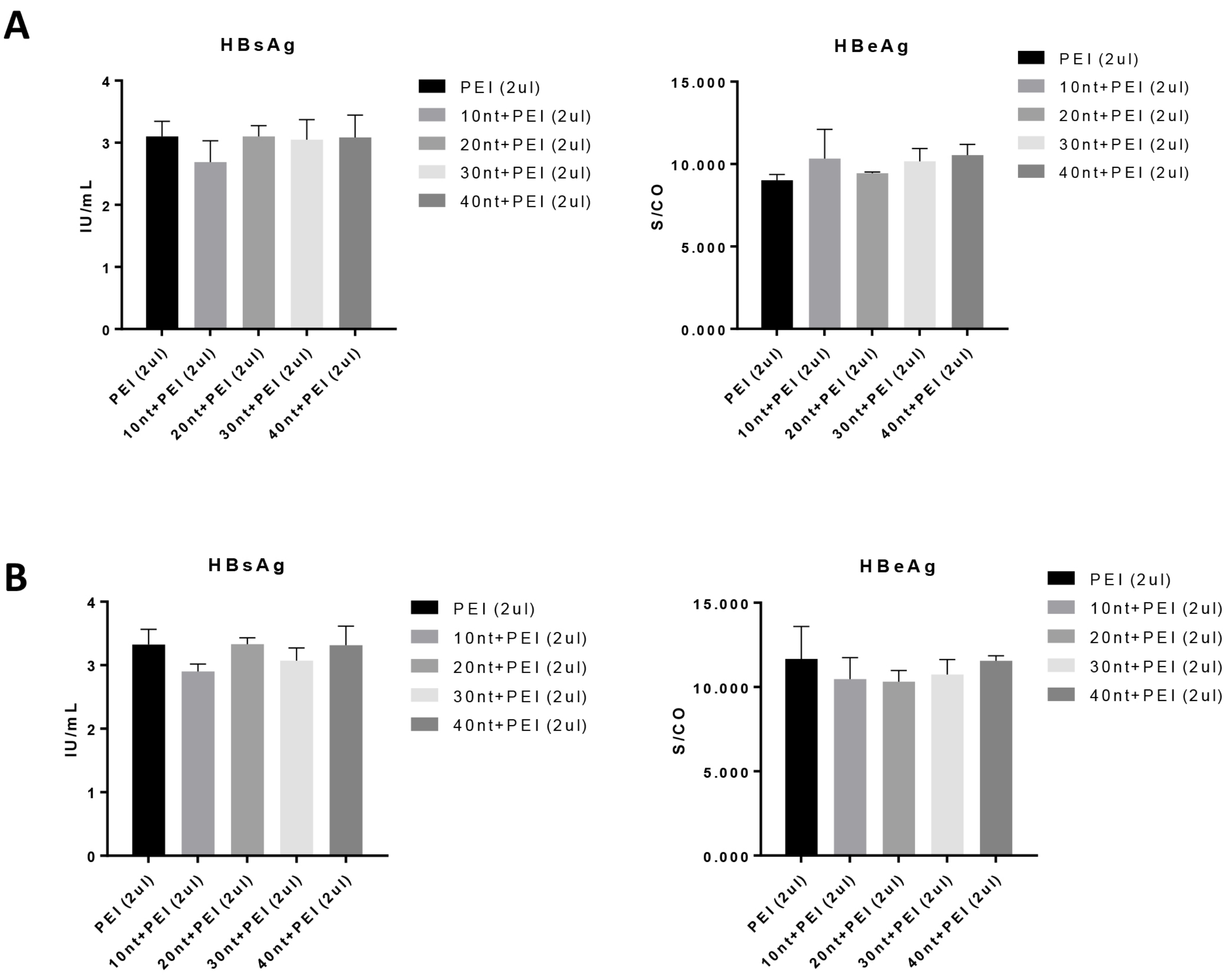

Supplementary Figure 2. The levels of HBsAg and HBeAg. HepAD38 cells cultured in 12-well plate were transfected with different length oligonucleotides using the commercial transfection reagents. (A) OneStep transfecter; (B) X-tremeGENE.
