# Supplementary material for: Transfection of unmodified oligodeoxynucleotide with polyethylenimine reduces the level of hepatitis B surface antigen
Source: Front Microbiol. 2025 May 1;16:1600679. doi: 10.3389/fmicb.2025.1600679 (PMC12078216; doi:10.3389/fmicb.2025.1600679)
Supplement: Supplementary file 3 [file Image_3.pdf]

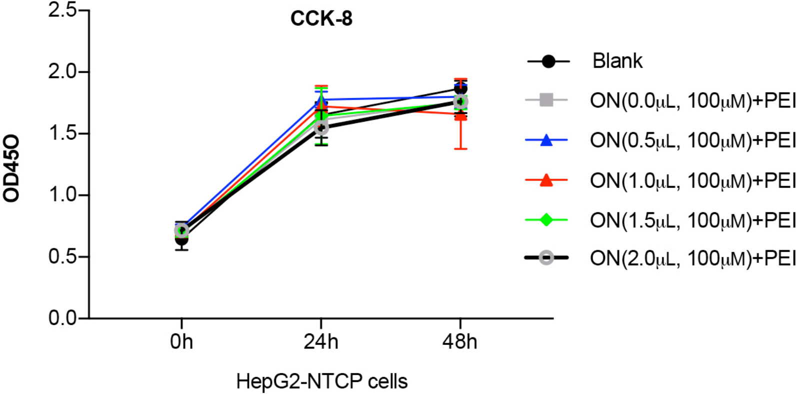

Supplementary Figure 3. The proliferation of HepG2-NTCP cells were measured by using the Cell Counting Kit-8 assay.
