# Supplementary material for: Transfection of unmodified oligodeoxynucleotide with polyethylenimine reduces the level of hepatitis B surface antigen
Source: Front Microbiol. 2025 May 1;16:1600679. doi: 10.3389/fmicb.2025.1600679 (PMC12078216; doi:10.3389/fmicb.2025.1600679)
Supplement: Supplementary file 5 [file Image_5.pdf]

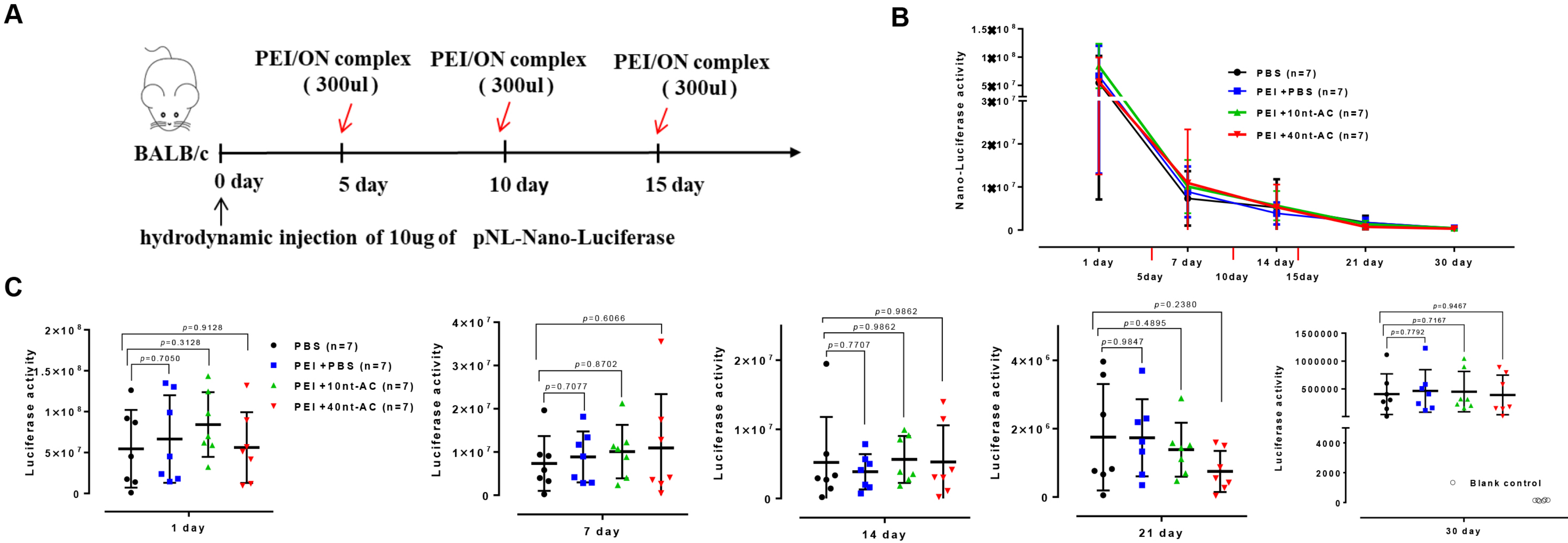

Supplementary Figure 5. (A)Schematic representation of the mice experiment. The 6-weeks-old BALB/c male mice were hydrodynamiclly injected with pNL-sNLuc plasmid through tail-vein at day 0. The injections of PBS, PEI, PEI/10nt-AC complex and PEI/40nt-AC complex are indicated by red arrows. (B), (C)Serum samples were collected at indicated time points and the luciferase activities of kinetics in mice were analyzed.
