# Supplementary figures and images for: Transfection of unmodified oligodeoxynucleotide with polyethylenimine reduces the level of hepatitis B surface antigen
Source: Front Microbiol. 2025 May 1;16:1600679. doi: 10.3389/fmicb.2025.1600679 (PMC12078216; doi:10.3389/fmicb.2025.1600679)

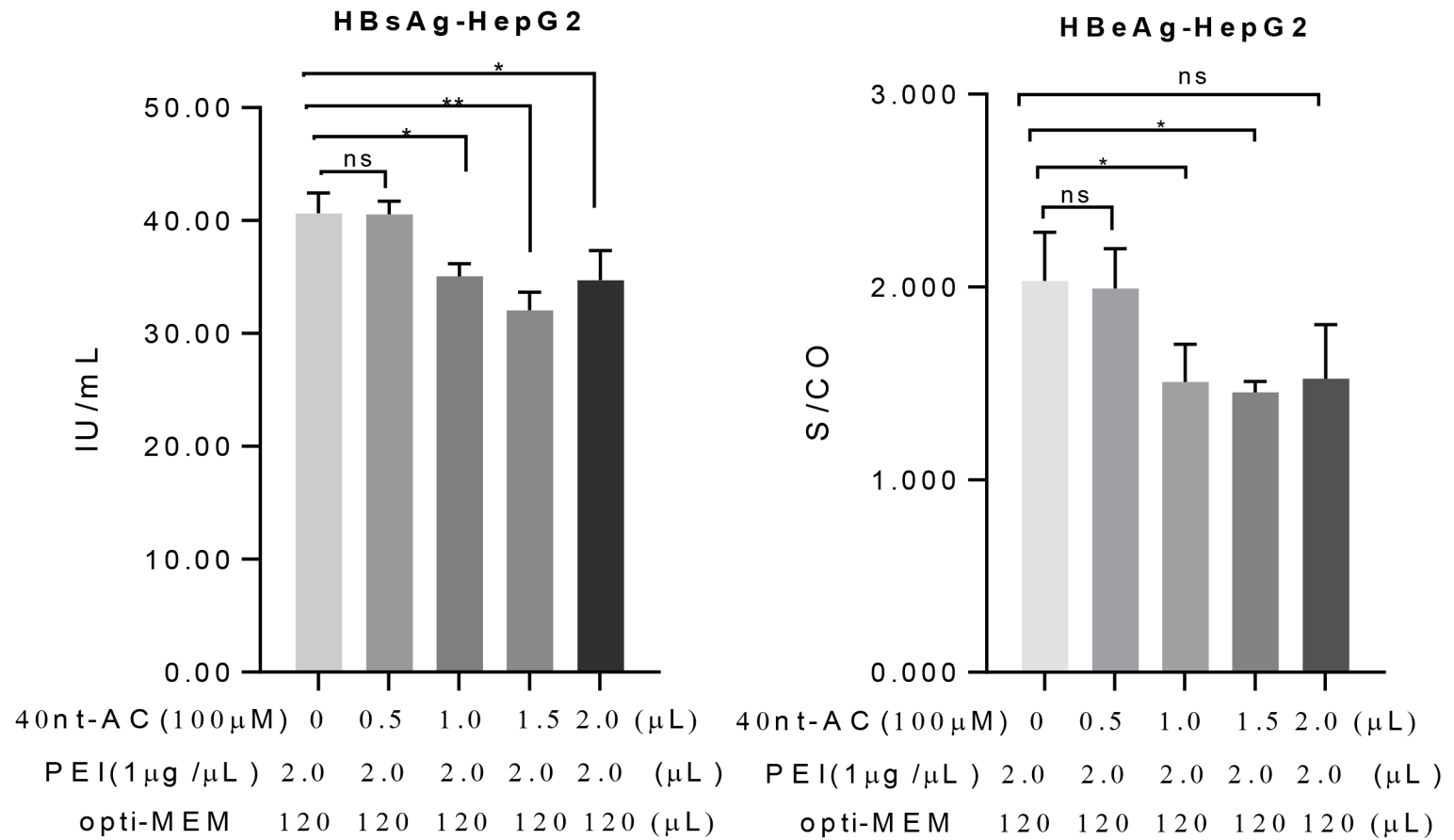

Supplement: Supplementary file 6 [file Image_6.pdf]
